# Aggresomes predict poor outcomes and implicate proteostasis in the pathogenesis of pediatric choroid plexus tumors

**DOI:** 10.1007/s11060-020-03694-3

**Published:** 2021-01-26

**Authors:** Nada Amer, Hala Taha, Dina Hesham, Nouran Al-Shehaby, Amal Mosaab, Mohamed Soudy, Aya Osama, Noura Mahmoud, Moatasem Elayadi, Ayda Youssef, Mohamed Elbeltagy, Mohamed Saad Zaghloul, Sameh Magdeldin, Ahmed A. Sayed, Shahenda El-Naggar

**Affiliations:** 1 Tumor Biology Research Program, Basic Research Unit, Research Department, Children’s Cancer Hospital Egypt, 1 Sekket El Emam, El Madbah El Kadeem Yard, Sayeda Zeinab, Cairo, 57357 Egypt; 2 Department of Pathology, Children’s Cancer Hospital Egypt, Cairo, 57357 Egypt; 3 Department of Pathology, National Cancer Institute, Cairo University, Cairo, Egypt; 4 Proteomics and Metabolomics Research Program, Basic Research Unit, Research Department, Children’s Cancer Hospital Egypt, Cairo, 57357 Egypt; 5 Department of Pediatric Oncology, Children’s Cancer Hospital Egypt, Cairo, 57357 Egypt; 6 Department of Pediatric Oncology, National Cancer Institute, Cairo University, Cairo, Egypt; 7 Department of Radiology, Children’s Cancer Hospital Egypt, Cairo, 57357 Egypt; 8 Department of Radiology, National Cancer Institute, Cairo University, Cairo, Egypt; 9 Department of Neurosurgery, Children’s Cancer Hospital Egypt, Cairo, 57357 Egypt; 10 Department of Neurosurgery, Faculty of Medicine, Cairo University, Cairo, Egypt; 11 Department of Radiotherapy, National Cancer Institute, Cairo University, Cairo, Egypt; 12 Department of Radiotherapy, Children’s Cancer Hospital Egypt, Cairo, 57357 Egypt; 13 Department of Physiology, Faculty of Veterinary Medicine, Suez Canal University, Ismailia, Egypt; 14 Genomics Research Program, Basic Research Unit, Research Department, Children’s Cancer Hospital Egypt, Cairo, 57357 Egypt; 15 Department of Biochemistry, Faculty of Science, Ain Shams University, Cairo, Egypt

**Keywords:** Aggresomes, Choroid plexus, Proteogenomics, Proteostasis, Prognosis

## Abstract

**Purpose:**

Protein misfolding and aggregation result in proteotoxic stress and underlie the pathogenesis of many diseases. To overcome proteotoxicity, cells compartmentalize misfolded and aggregated proteins in different inclusion bodies. The aggresome is a paranuclear inclusion body that functions as a storage compartment for misfolded proteins. Choroid plexus tumors (CPTs) are rare neoplasms comprised of three pathological subgroups. The underlying mechanisms of their pathogenesis remain unclear. This study aims to elucidate the prognostic role and the biological effects of aggresomes in pediatric CPTs.

**Methods:**

We examined the presence of aggresomes in 42 patient-derived tumor tissues by immunohistochemistry and we identified their impact on patients’ outcomes. We then investigated the proteogenomics signature associated with aggresomes using whole-genome DNA methylation and proteomic analysis to define their role in the pathogenesis of pediatric CPTs.

**Results:**

Aggresomes were detected in 64.2% of samples and were distributed among different pathological and molecular subgroups. The presence of aggresomes with different percentages was correlated with patients’ outcomes. The ≥ 25% cutoff had the most significant impact on overall and event-free survival (p-value < 0.001) compared to the pathological and the molecular stratifications.

**Conclusions:**

These results support the role of aggresome as a novel prognostic molecular marker for pediatric CPTs that was comparable to the molecular classification in segregating samples into two distinct subgroups, and to the pathological stratification in the prediction of patients’ outcomes. Moreover, the proteogenomic signature of CPTs displayed altered protein homeostasis, manifested by enrichment in processes related to protein quality control.

**Supplementary Information:**

The online version contains supplementary material available at 10.1007/s11060-020-03694-3.

## Introduction

Choroid plexus tumors (CPTs) are rare intraventricular neoplasms that account for 0.2% to 0.4% of all central nervous system neoplasms, with up to 20% during the first year of life [1]. CPTs are pathologically classified into three subgroups; benign choroid plexus papilloma (CPP, WHO grade I), intermediate atypical CPP (ACPP, WHO grade II), and the aggressive malignant choroid plexus carcinoma (CPC, WHO grade III) that is associated with poor prognosis [2]. CPPs have a favorable prognosis after surgical resection and rarely require additional treatment [3], while CPCs usually require surgical removal with adjuvant chemotherapy and radiotherapy [3]. The molecular stratification of pediatric CPTs identified two subgroups that distinguished high-risk patients regardless of their pathological classification [4, 5]. Current evidence supports the involvement of TP53 [4], Notch signaling [6], and Sonic Hedgehog (SHH) [7] in the pathogenesis of these tumors; however, the underlying etiology is yet to be identified.

The cellular proteome is a highly complex system that requires the coordination of protein quality control (PQC) machinery to balance protein synthesis, folding, and degradation [8]. Continual proteotoxic stress imposed by an imbalance in protein levels or protein aggregation causes cells to compartmentalize misfolded/ aggregated proteins into distinct quality control compartments [8]. The aggresome is a single juxtanuclear inclusion body wherein misfolded proteins are delivered by the dynein-mediated retrograde transport and ensheathed by the intermediate filament vimentin [9]. Aggresome formation has emerged as a drug resistance mechanism to overcome proteotoxic stress caused by proteasome inhibition-based therapy in selective tumors such as multiple myeloma [10, 11], pancreatic cancer [12], breast cancer [13], and lymphoma [10]. We have previously identified aggresomes in pediatric CPTs [14] and pediatric medulloblastoma (MB) [15]. They were inherently present in both tumors before therapy and were associated with poor outcomes in the non-WNT/SHH molecular subgroup of MB [15], suggesting that they provide a survival advantage to these tumors.

In the current study, we report the role of aggresomes as a prognostic molecular marker in pediatric CPTs. Additionally, we explore the molecular signature of aggresome-positive CPTs using genome-wide methylation profile in correlation to cellular proteome which implicates altered proteostasis in the pathogenesis of pediatric CPTs.

## Materials and methods

### Patient and tissue samples

CPT samples were retrospectively collected from the Pathology Department at the Children’s Cancer Hospital Egypt 57357 (CCHE) after the approval of the Institutional Research Ethics Board (IRB) for waiver of consent. None of the patients had previously received chemotherapy or radiotherapy. Histopathologic review of all CPT samples was performed according to the WHO guidelines. Our cohort was comprised of 23 patient formalin-fixed-paraffin embedded (FFPE) tissue samples, 2 fresh frozen (FF), and 17 patients represented by both FFPE and their matched FF tissue samples. Treatment protocol of CPT patients was adopted from CPT-SIOP-2009 study [16].

### Immunohistochemical analysis (IHC)

IHC was performed using the Ventana Benchmark XT automated system (Ventana Medical System). Antibodies against the following antigens were used: TP53 (Clone DO-7, N1581, Dako; ready to use), vimentin (Ventana 790-2917; dilution 1:100), and pan-keratin (Ventana 760-2595; dilution 1:100). Aggresome-positivity was considered only for cells exhibiting juxtanuclear staining of vimentin.

### Sample processing and the Infinium methylation EPIC array

Genomic DNA was extracted from FFPE tumor samples using QIAamp DNA FFPE tissue kit (Qiagen) and FF tumor samples using the Gene JET genomic DNA (Thermo Fisher) according to the manufacturer’s instructions. DNA was quantified using the DENOVIX Fluorometer (ds DNA High Sensitivity). The quality of the extracted FFPE DNA samples was assessed by Illumina FFPE QC kit (Illumina Inc.). Bisulfite conversion of extracted DNA was performed using the EZ DNA methylation kit (D5002, Zymo Research) according to the manufacturer’s instructions using the alternative incubation conditions recommended for the Illumina Infinium methylation arrays. Bisulfite-converted FFPE DNA was then restored with Infinium HD FFPE DNA Restore Kit (WG-321-1002, Illumina Inc.). Restored bisulfite-modified DNA samples were hybridized to the Illumina Infinium Human Methylation EPIC bead chips and scanned using the Illumina iScan microarray scanner according to the manufacturer’s recommendations (Illumina Inc.). Methylation data are available through Gene Expression Omnibus (GEO: http://www.ncbi.nlm.nih.gov/geo/), accession number GSE156090.

### Methylation data analysis

Methylation analysis was performed using R statistical language v.3.5.2. Raw signal intensities were obtained from Illumina intensity data (IDAT) files using the minfi Bioconductor package v. 1.29.3 [17]. Samples quality control steps were performed including; sample swap using the pairwise comparison of 59 genotyping probes and detection of *p-value*. Samples with *p-value* > *0.01* (n = 2) were excluded from further analysis. Each sample was individually normalized using functional normalization (FunNorm) [18]. Batch effect prediction was done by singular value decomposition (SVD) using the ChAMP Bioconductor package v.3.10 [19], subsequent correction for the type of tumor samples (FFPE or FF) was performed by ComBat algorithm [20]. Probes failed in the detection of *p-value* > *0.01* in at least one sample (n = 91,679) and probes located on sex chromosomes (n = 15,413) were removed. Probes containing SNPs (n = 24,747) in their bodies, or at a CpG, or single base extension site (SBE) with minor allele frequency (maf) ≥ 0.01, were excluded and those showing cross-reactivity (n = 38,077) [21, 22] were eliminated. Beta (β) and M values were generated from the remaining probes (n = 696,293). The most variably methylated probes (n = 36,279) were selected based on the standard deviation (SD > 0.7). The t-distributed stochastic neighbour embedding (t-SNE) analysis was performed using Rtsne package v.0.11 [23] with non-default parameters [theta = 0, pca = F, max_iter = 2000] based on pairwise Pearson’s correlation. Unsupervised hierarchical clustering was done using Euclidean distance and ward.D2. The next-generation molecular neuropathology (MNP) platform was used to check the methylation-based classification of our CPT samples [24]. All duplicates samples (n = 17) were removed from any further analysis. Differentially methylated positions (DMPs) were identified using champ.DMP function from ChAMP Bioconductor package v.3.10 [25] at the significance of adjusted *p-value* < *0.05*. Differentially Methylated Regions (DMRs) were identified by Bumphunter with default settings [26]. Circos plot of DMRs was performed by Circlize package v0.4.8 with default parameters [27]. Three stratification approaches of CPTs (methylation-based classification, aggresome-positivity, and the ≥ 25% aggresome cutoff) were used to examine the correlation of aggresomes with methylation signature.

### Methylation data GO and KEGG enrichment analyses

The gene lists derived from DMPs with adjusted *p-value* < *0.05* and log FC (1.5, − 1.5) were used for gene set enrichment analysis using ToppFun in the ToppGene suite [28]. Genomic Regions Enrichment of Annotations Tool v3.0.0 (GREAT) was utilized for the gene ontology (GO) enrichment analysis of the DMRs using default settings [29]. Bed files denoting the start, the end positions of the DMRs, as well as the chromosome numbers were uploaded and mapped against the hg19 human reference genome. Network analysis of the DMPs’ genes was performed by NetworkAnalyst 3.0 using STRING interactome with 700 confidence score cutoff, and experimental evidence criterion was required [30]. Analyses for enriched Kyoto Encyclopedia of Genes and Genomes (KEGG) pathways of those networks were performed. Only terms with false discovery rate (FDR) less than 0.05 using the Benjamini-Hochberg (BH) method were considered significant for all analyses.

### Survival analysis

All statistical analyses were performed using R statistical environment v3.3.2. Overall survival (OS) was calculated from the initial diagnosis to last follow-up or death due to disease. Event-free survival (EFS) was calculated from the initial diagnosis to the time of an event. An ‘event’ was defined as tumor progression, recurrence or death. OS and EFS were estimated by the Kaplan–Meier method, and differences between groups were assessed by the log-rank test. Survival estimates referred to 2 years from diagnosis and the related 95% confidence intervals (95% CI) were calculated. One patient died shortly after surgery before assigning any protocol and was removed from the analysis.

### Protein extraction

Twenty one frozen samples were collected and sample sections were homogenized in urea extraction buffer (8 M urea, 500 mM Tris–HCl pH 8.5, and protease inhibitors) using Dounce homogenizer. Lysates were incubated at room temperature for 1 h before centrifugation at 10,000 rpm for 30 min. Supernatants containing extracted proteins were collected and proteins were quantified using the Pierce BCA protein assay kit (23225, Thermo Fisher).

### In-gel digestion

Forty micrograms of proteins from each sample were separated using 12% SDS-PAGE. Proteins were fixed by adding a fixing solution (50% methanol and 12% acetic acid) with overnight incubation at 4 °C. Gel pieces were washed using gel-wash buffer (50% acetonitrile in 50 mM ammonium bicarbonate) and dried using speed vacuuming. Reduction buffer (10 mM dithiothreitol in 50 mM ammonium bicarbonate) was added to dried gel pieces and incubated at 60 °C for 30 min. Alkylation buffer (55 mM iodoacetamide in 50 mM ammonium bicarbonate) was then added and incubated in dark at room temperature for 30 min. Gel pieces were washed using 25 mM ammonium bicarbonate before adding acetonitrile for 15 min. Digestion solution (10 ng/μL trypsin in 25 mM ammonium bicarbonate) was added to gel pieces until gel hydration and incubated overnight at 37 °C. Extraction buffer (66 acetonitrile: 33 milliQ water: 1 formic acid) was then added to extract digested peptides.

### LC–MS/MS

The nanoflow reverse-phase liquid chromatography (LC) followed by mass spectrometry (MS/MS) analysis was carried out using Triple TOF 5600 + (AB SCIEX) interfaced at the front end with an Eksigent nano-LC 400 auto-sampler with an Ekspert nano-LC 425 pumps. Samples were automatically injected into a peptide trap column Chrome XP; C18-CL, 5 µm (Chrome XP; C18-CL, 0.5 mm I.D. × 10 mm, 5-μm particle size, 120-Å pore size; SCIEX). The MS and MS/MS ranges were 400–1250 m/z and 170–1500 m/z, respectively. A 55-min linear gradient of 3–40% solution B (80% acetonitrile, 0.2% formic acid) was applied. The mass spectrometry proteomics data have been deposited to the ProteomeXchange Consortium via the PRIDE [1] partner repository with the dataset identifier PXD021076 and 10.6019/PXD021076. RAW files were converted into mascot generic format (mgf) using AB-SCIEX MS data converter v.1.3 and searched using X!Tandem in peptide shaker v. 1.16.38 against human UniProtKB/Swiss-Prot database (2018 release; 173361 proteins). Proteins identified with a minimum of one unique peptide and a minimum of two confident spectra were selected for further statistical analyses.

### Proteomics data analysis

Twenty one exported CSV files containing Uniprot accession number and label-free protein abundance based on normalized spectral abundance factor (NSAF) were merged into a single file using ProteoSelector (www.57357.org/proteomics-unit). A sanity check was performed to evaluate the accuracy of the sample, class labels, and data structure. Then the data imputation was conducted based on the minimum NSAF protein value [31] followed by the filtration process based on non-relative standard deviation (NRSD) [32]. Samples were then normalized using probabilistic quotient normalization (PQN) [33] then log-transformed, and auto-scaled. Data was subjected to unpaired t-test and the significant output was considered only when p-value ≤ 0.05. The significant differentially expressed proteins (DEPs) were analyzed using UniProtR [34]. The biological processes, molecular functions, and cellular components of identified protein were obtained by ToppFun in the ToppGene suite [28]. Protein–protein interactions (PPI) and network analyses were carried out by Cytoscape [35].

## Results

### Aggresome predicts poor prognosis CPT patients

**Fig. 1:**
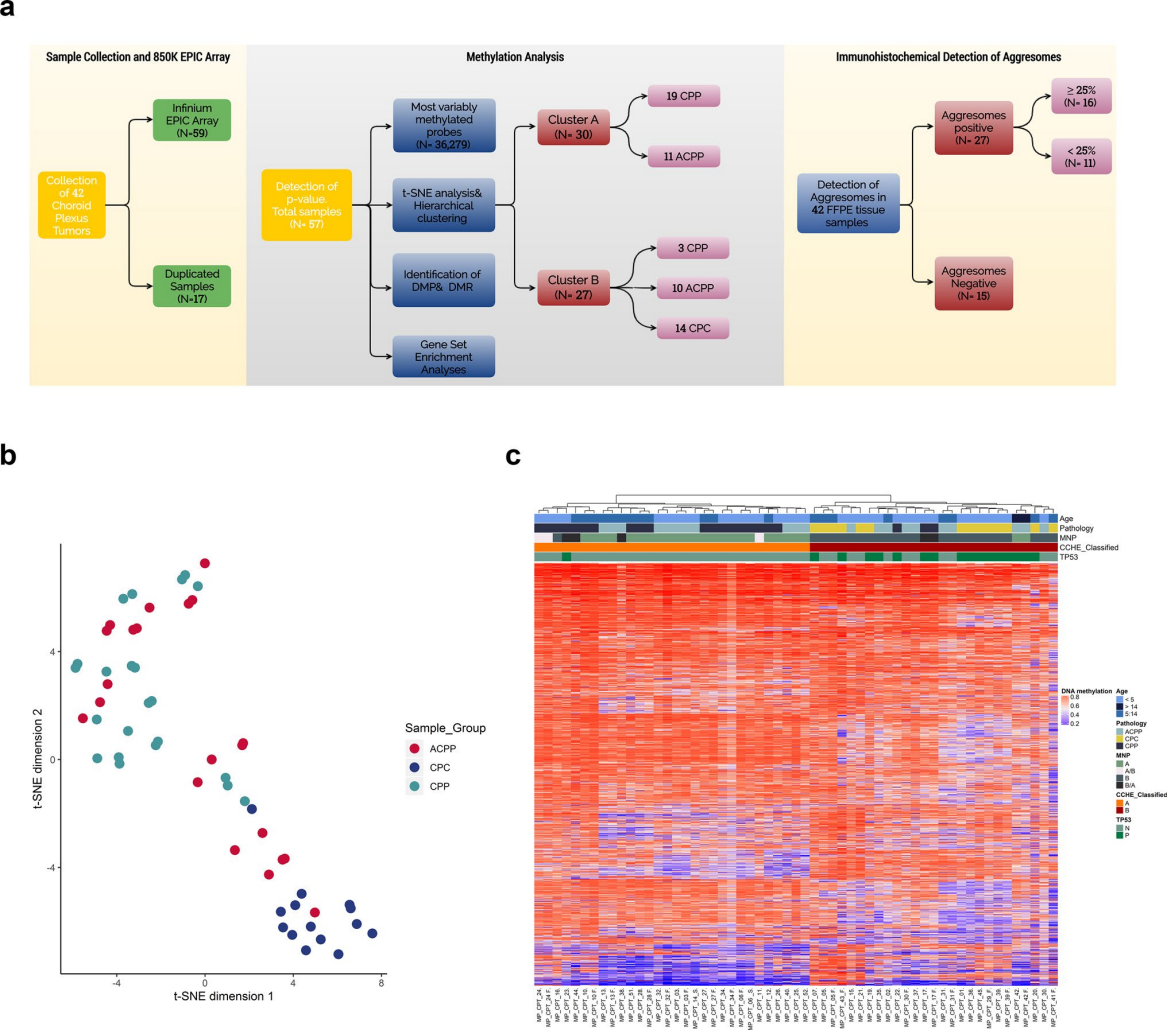
Methylation-based classification of pediatric CPTs identified two molecular subgroups. **a** Overview flow chart of sample population and analyses. **b** t.SNE plot of the CPT dataset (n = 57) using the most variable methylated probes (n = 36,279), showing two distinct molecular entities compared to pathological classification. **c** Heatmap of β-values of the most variable methylated probes after the assignment of CPTs subgroups into clusters "A" and "B", combined with age, pathology, MNP classification, and TP53 status

The clinicopathological characteristics of all patients are summarized in (Supplementary Table 1). Methylation EPIC array was used to molecularly stratify CPT samples (Fig. 1a). Sample swap affirmed the concordance between matched FFPE and FF tissue samples (Supplementary Fig. 1a). Two samples were excluded from further analysis based on a high *p-value* compared to background signals of control probes (Supplementary Fig. 1b). Technical batch effects were excluded by singular value decomposition (SVD) (Supplementary Fig. 1c). Normalized and filtered data of 57 tumor samples were used for the selection of the most variable methylated probes (n = 36,279). These probes were distributed across all chromosomes and different genomic features, with the majority in the open sea and gene bodies (Supplementary Fig. 1d). Subsequent t-SNE analysis and hierarchical clustering across the dataset using most variable probes identified two distinct groups with all paired samples of FFPE and FF clustered together (Fig. 1b and Supplementary Fig. 2). Methylation cluster “A” comprised 19 CPPs (63.33%) and 11 ACPP (36.67%.) with no CPC samples. In contrast, cluster “B” contained all CPC samples (n = 14, 51.8%), 3 CPPs (11.11%), and 10 ACPP (37.09%). The MNP classifier was used to validate the methylation-based classification of CPT samples (Fig. 1c and Supplementary Table 2).

**Fig. 2:**
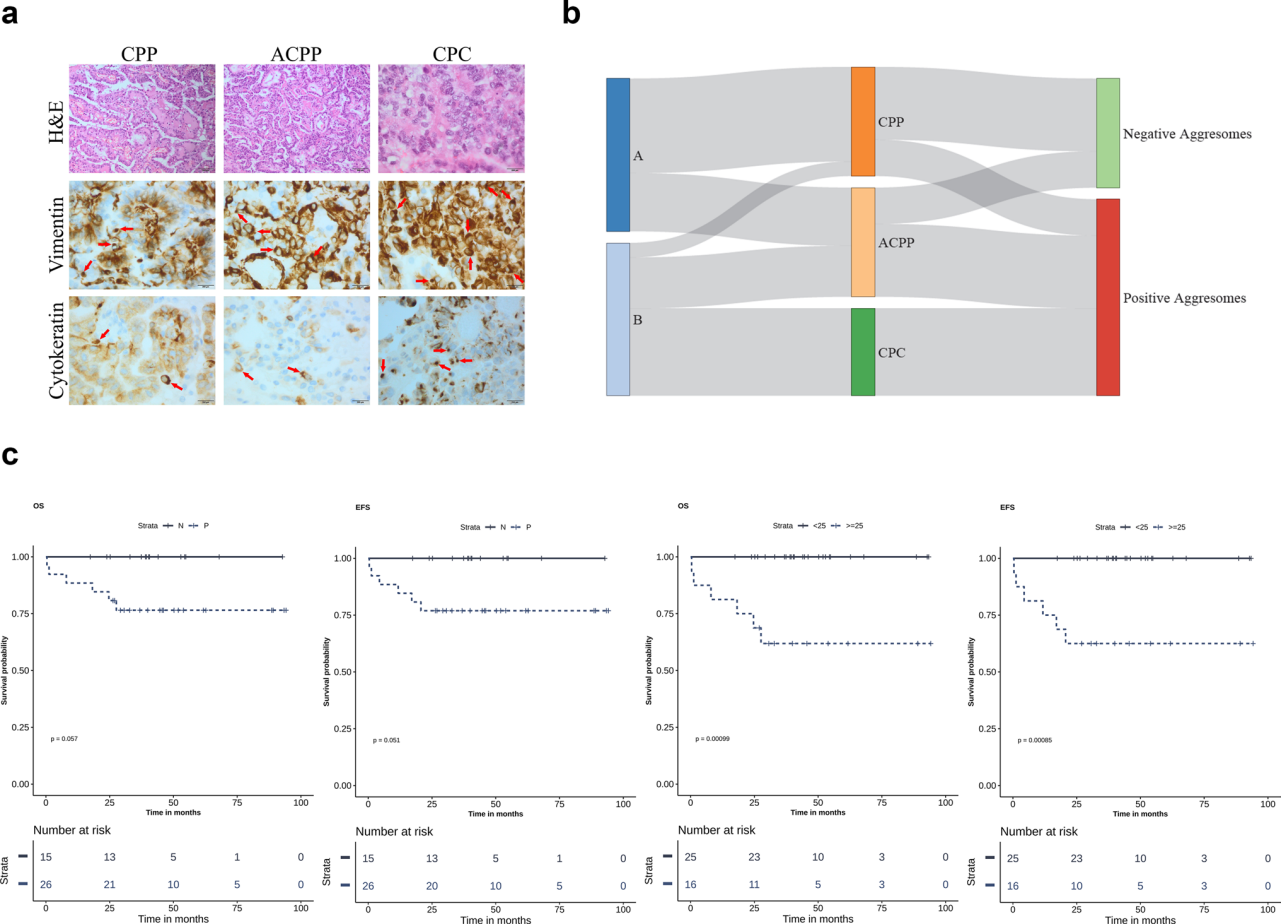
Characterization of aggresomes in CPTs and their association with the clinical outcomes. **a** Hematoxylin and eosin (H&E) staining of CPP, ACPP, and CPC FFPE tissues and IHC analysis of vimentin and cytokeratin. Juxtanuclear dot-like staining of vimentin and cytokeratin were detected in all pathological subgroups with different percentages. **b** Distribution of aggresomes among different pathological and molecular subgroups; NetworkD3 and dplyr packages were used to obtain the Sankey plot. **c** Kaplan–Meier plot of overall (OS) and event-free survival (EFS) for 41 CPT patients. Survival analysis was separated by aggresome-positivity (positive vs. negative), and the ≥ 25% aggresomes cutoff. *P-values* were calculated using the log-rank test

IHC identified aggresomes in 64.2% of samples (n = 27) distributed among all pathological and molecular subgroups. The cytokeratin-positive paranuclear stain was detected in most of the samples, however; its percentage did not coincide with the level of vimentin (Fig. 2a and Supplementary Table 2). Aggresomes-based classification identified two groups, where aggresome-positive tumors comprised all CPCs (n = 12), ACPPs (n = 10), CPPs (n = 5), and 77.7% of cluster “B” tumors (n = 21). On the other hand, CPPs (n = 10), ACPPs (n = 5), and 71.4% of cluster “A” tumors (n = 15) fell in the aggresome-negative group (Fig. 2b). The overexpression of TP53 was observed in 26.19% of all tumors (n = 11). TP53-positive tumors all had aggresomes, fell in cluster “B” and distributed among all pathological subgroups (Supplementary Table 2). TP53 had a significant impact on both OS and EFS (*p-value* = *0.03*) (Supplementary Fig. 3). In addition, the proliferation indices of Ki-67/MIB-1with ≥ 30% cutoff aslo had a significant effect on OS and EFS; *p-value* < *0.0001*) (Supplementary Table 3). Survival analysis using different percentages of aggresomes ranging from 10 to 30% was performed to test the impact of aggresome on patient outcome compared to pathological and molecular classifications, and TP53 status (Supplementary Table 4 and Supplementary Fig. 3). Aggresome-positivity correlated significantly with patients’ outcomes and maintained significance at all proposed cutoffs. The ≥ 25% aggresomes cutoff had the highest impact on both OS and EFS (*p-value* < *0.001*) (Fig. 2c). Analysis of clinical variables including age at diagnosis (OS *p-value* = *0.53* and EFS; *p-value* = *0.54*), and extent of resection (OS and EFS; *p-value* = *0.97*) had no significant effect on outcomes (Supplementary Table 3).

### DNA methylation signature implicates proteostasis in the pathogensis of CPTs

**Fig. 3:**
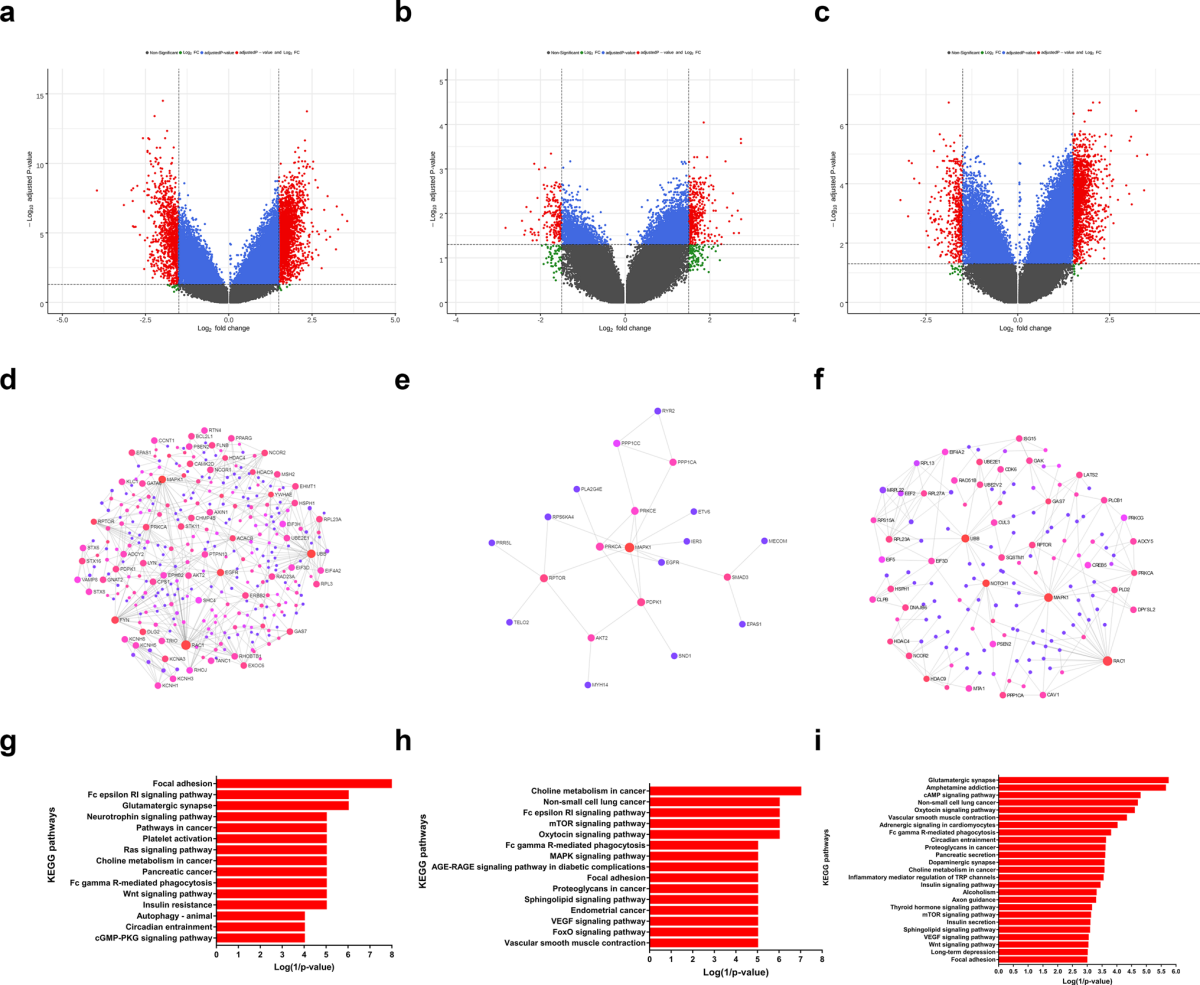
Enrichment analysis of DMPs. Volcano plot of the DMPs highlighting the hypomethylated and hypermethylated genes in **a** methylation-based classification, **b** aggresomes-positivity, and **c** the ≥ 25% aggresomes cutoff, with log2 FC on the x-axis and log10 adjusted *p-value* on the y-axis. The horizontal line represents the cutoff of the adjusted (*p-value* < 0.05) and the vertical lines represent the cutoff of the log2 FC (1.5 and -1.5). PPI network analysis of significant DMPs of **d** methylation-based classification, **e** aggresomes-positivity, and (f) the ≥ 25% aggresomes cutoff. Enrichment analysis of KEGG pathways in **g** methylation-based classification, **h** aggresomes-positivity, and (i) the ≥ 25% aggresomes cutoff with enriched pathways plotted on the y-axis versus their Log (1/*p-values*) on the x-axis

Statistically significant DMPs of the three stratification approaches were identified (Supplementary Tables 5 and 6) and used for gene set enrichment analysis (GSEA) (Supplementary Table 7). Methylation-based classification using DMPs with adjusted *p-values* < *0.05* and log FC (1.5, −1.5) identified 1840 genes that showed enrichment in developmental processes like regulation of cell morphogenesis, neuron development and generation in addition to regulation of protein localization, and cytoskeleton protein binding organization (Fig. 3a and Supplementary Fig. 4a). Aggresome-positivity identified 358 genes that had a functional enrichment of protein binding and microfilament motor activity, as well as intracellular protein transport and localization (Fig. 3b and Supplementary Fig. 4b). Furthermore, the 25% aggresomes cutoff comparison identified 1243 genes enriched in developmental processes, protein binding, voltage-gated ion channel, and protein localization (Fig. 3c and Supplementary Fig. 4c).

PPI network of aggresome-positivity was included within the methylation-based classification network (Fig. 3d, e, f, and Supplementary Table 8). The PPI networks of the differentially methylated genes of the three comparisons were enriched in the mTOR signaling pathway, MAPK, autophagy, apoptosis, and PI3K-Akt signaling pathway, mitophagy, pancreatic, prostate, and breast cancers in addition to chronic myeloid leukemia pathways (Fig. 3g, h, and i). Despite having the lowest number of genes in the PPI subnetwork, aggresomes positivity identified the highest number of enriched pathways with a 30.8% concordance with methylation-based classification (Supplementary Fig. 4d).

**Fig. 4:**
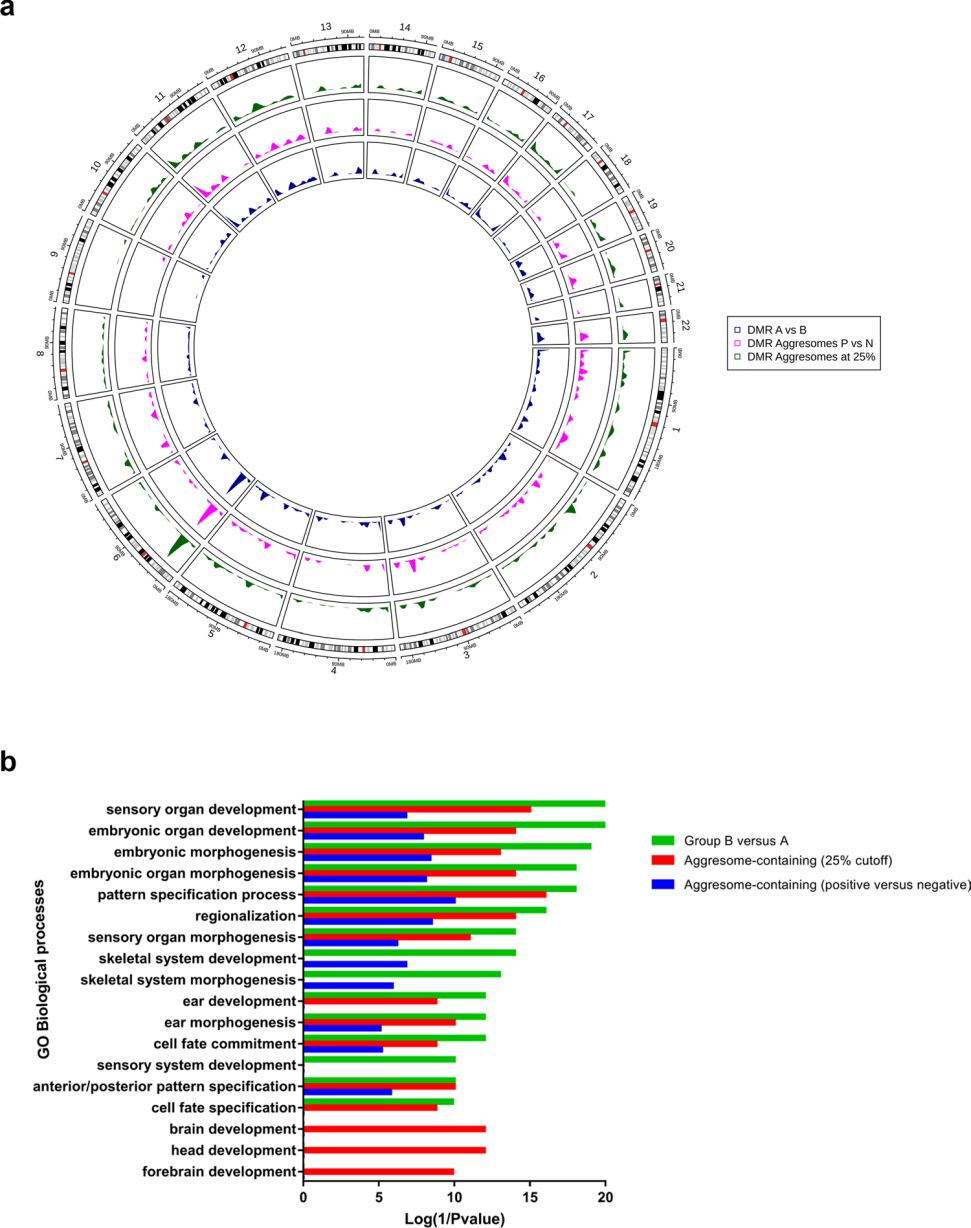
DMRs and their genomic context. **a** Circos plot of DMRs for the 3 comparisons and their distributions across chromosomes. **b** GO analysis of top significant (FDR < 0.05) biological processes, molecular functions, and cellular components of DMRs in all each stratification approaches

DMRs of the three stratification approaches displayed a similar pattern across all chromosomes (Fig. 4a and Supplementary Table 9). *ZIC1* and *ZIC4* genes were identified in the top significant DMRs in the three stratification approaches (*p-values* < 0.000). DMPs that annotated to those genes were found in the body and promoter regions and were hypermethylated in all positions and all comparisons. The GO analysis of DMRs mainly showed enrichment in developmental processes (Fig. 4b and Supplementary Table 10). Furthermore, pathway enrichment analysis identified, the intrinsic apoptotic signaling pathway by p53 class mediator pathway, and the ATF6-mediated unfolded protein response (Supplementary Table 10).

### Proteomics support DNA methylation signature in CPTs

**Fig. 5:**
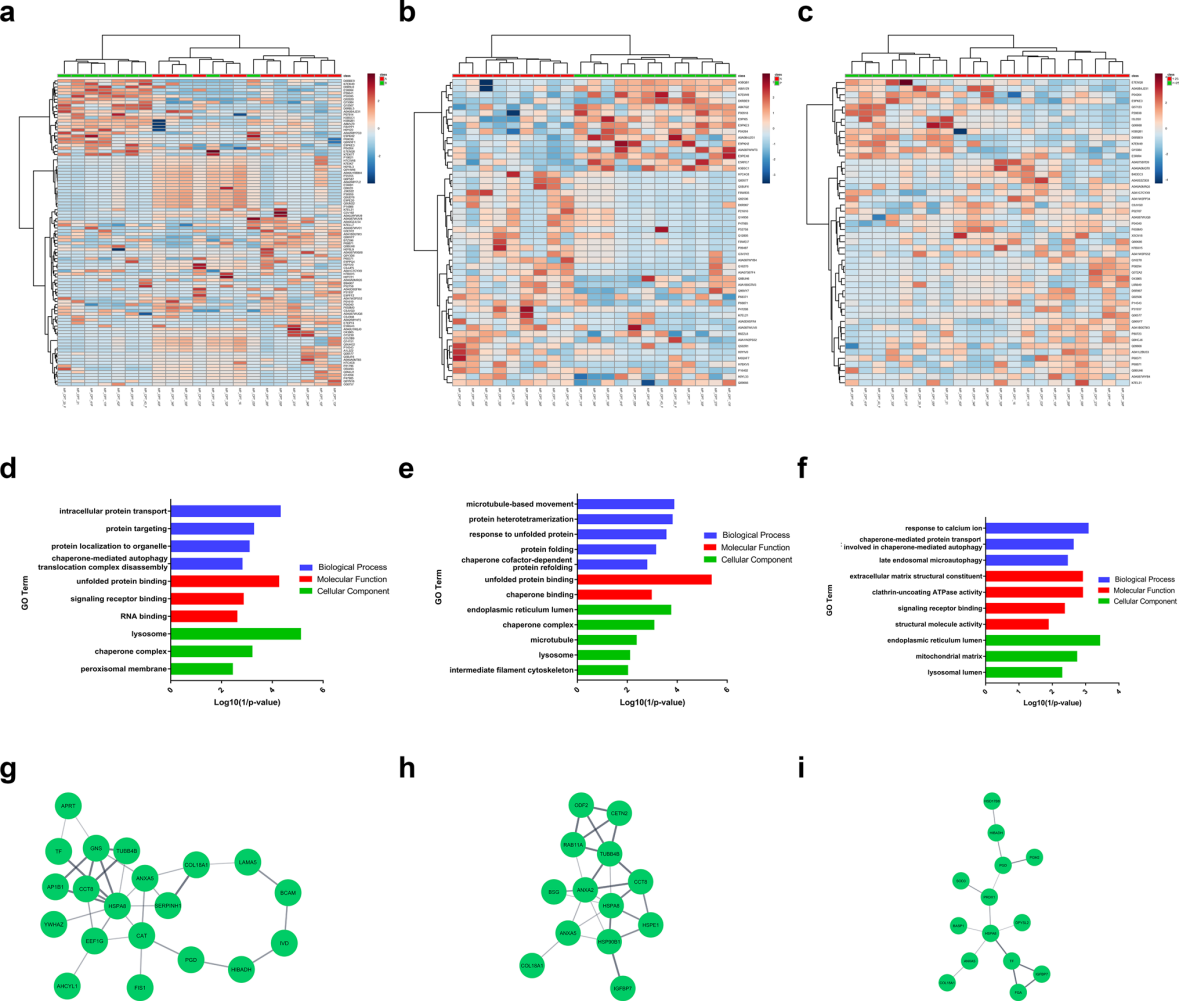
Proteomic signature of CPTs. Hierarchical clustering heatmap of the top 50 significant DEPs (*p-values* < *0.05*) of **a** methylation-based classification, **b** aggresomes-positivity, and **c** the ≥ 25% aggresomes cutoff, with the y-axis represents samples analyzed and the x-axis denotes Uniprot accessions. GO enrichment analysis of DEPs of **d** methylation-based classification, **e** aggresomes-positivity, and **f** the ≥ 25% aggresomes cutoff. PPIs of interacting DEPs in **g** methylation-based classification, **h** aggresomes-positivity, and **i** the ≥ 25% aggresomes cutoff

A total of 2147 proteins were identified from all samples, filtered into 784 non-redundant proteins for subsequent normalization and differential analysis (Supplementary Table 11). DEPs among the three stratification approaches were identified (Supplementary Fig. 5a, b, c, and Supplementary Table 12) and segregated CPT samples into two distinct subgroups using partial least square regression analysis (PLS) (Supplementary Fig. 5d, e, and f). Hierarchical clustering using the top 50 DEPs showed a clear separation between samples specifically within the aggresome-positivity stratification (Fig. 5a, b, and c).

The GO analysis of the DEPs derived from methylation-based classification showed enrichment in terms of protein localization and transport, chaperone-mediated autophagy, unfolded protein response, RNA binding, and lysosomal degradation (Fig. 5d, Supplementary Table 13). DEPs of aggresome-positivity showed enrichment in the cellular response to unfolded protein along with chaperone binding, microtubule-based movement, and intermediate filament remodeling (Fig. 5e, Supplementary Table 13). Finally, DEPs based on the 25% aggresomes cutoff showed enrichment in cellular processes involved in late endosomal microautophagy, chaperone-mediated autophagy, lysosomal degradation, and calcium homeostasis (Fig. 5f, Supplementary Table 13). The PPI network of DEPs in the three stratifications were found to be differentially methylated genes and related to protein regulation machinery (Fig. 5g, h, and i).

## Discussion

In this study, we assessed the prognostic impact of aggresomes in pediatric CPTs and examined their associated proteogenomic signatures. CPT patients were molecularly stratified using genome-wide methylation profiling. Consistent with previous reports [4, 5] the methylation-based classification of pediatric CPTs identified two molecular subgroups with all CPCs clustered in one subgroup, while ACPPs and CPPs distributed among both subgroups. The molecular stratification of CPTs had a significant impact on patient prognosis; however, the pathological stratification maintained the highest significance on patient outcomes. Despite having a lower significance than pathology, molecular classification identified ACPPs and CPPs with different biologic features similar to CPCs. The ACPPs are a challenging entity compared to CPPs and CPCs subgroups. Therefore, methylation-based classification provided a mean to segregate atypical tumors into good outcome (group A) or poor outcome (group B). In agreement with studies by other group [4, 5], methylation-based analysis was the most appropriate way to make the distinction. The overexpression of TP53 protein was observed only in the "B" molecular subgroup regardless of the pathological subtypes. Meanwhile, aggresomes were detected in all pathological and molecular subgroups with different percentages from negative up to 90% of tumor cells. The presence of aggresome had a nearly significant impact on the patient's outcome, while the ≥ 25% cutoff was comparable to the molecular classification in segregating samples into two subgroups, and to the pathological stratification in the prediction of patient outcomes. These results coincided with our previous study where a ≥ 20% aggresomes cutoff was a predictor of poor prognosis in the non-WNT/SHH molecular subgroup of pediatric MB [15]. The most commonly used model for assessing prognostic variables is multivariate survival analysis. Thereby, we calculated the minimum sample size required for a multivariable proportional hazards model using the significant prognostic variables based on univariate analysis (pathology, methylation-based classification, ≥ 25% aggresomes cutoff, the status of TP53, and the Ki67/MIB) per criteria proposed by Riley et al. [36]. Assuming estimated adjusted Cox-Snell R2 = 0.55, a minimum sample size of 71 patients with at least 15 outcome events is needed. Hence, our cohort was not powered for such analysis owing to the scarcity of CPTs, especially in a single center.

To further explore the molecular signature of pediatric CPTs and examine the correlation of aggresomes with genome-wide alterations we identified DMPs associated with aggresome formation in reference to the molecular subgroups. Various numbers of DMPs were associated with the three comparisons. The least number of DMPs in aggresome-based classification could be attributed to the fact that this comparison was based on a single biological feature; aggresomes. While the 25% cutoff applied more stringent criteria that increased the pathological and molecular uniformity of tumor populations. Interestingly, the methylation signature displayed by aggresome-positivity or the 25% aggresomes cutoff was the same as the methylation-based classification. Most of the enriched pathways were related to p53 binding, protein binding, folding, localization, or degradation in addition to the autophagy-related pathways such as mTOR signaling pathway and chaperone-mediated autophagy. Sholler et al. showed an activation of mTOR pathway in a chemo-resistant CPC patient and it was chosen for targeted therapy [37]. Therefore, further investigation of mTOR pathway in CPTs is warranted. Other pathways were enriched in DMPs including; pancreatic, colorectal, prostate, breast cancers as well as chronic and acute myeloid leukemias. All of these tumors had been reported to have aggresomes [10, 12, 13, 38–40], which implicates protein quality control in CPT pathogenesis. The manifestation of altered PQC signature that associated with DMPs was further confirmed by the enriched ATF6-mediated unfolded protein response in the DMRs. The enrichment analysis of DMRs revealed the TP53 signaling pathway which known to be involved in the development of CPTs [6, 37, 41, 42]. The top significant DMR in the three comparisons comprised of *ZIC1* and *ZIC4* genes. Interestingly, DMPs annotated to those genes were hypermethylated in the “B” molecular subgroup and aggresomes positive tumors with positions either at the promoter sites or in the body of the gene. *ZIC* family genes are involved in a variety of developmental processes, including neurogenesis and morphogenesis [43]. Recently, *ZIC1* gene was found to be silenced in colon cancer cell lines [44], primary colorectal cancer tissues [44], and gastric cancer [45]. Ectopic expression of ZIC1 suppressed cell proliferation and induced apoptosis through the MAPK and PI3K/Akt pathways, as well as the Bcl-xl/Bad/Caspase3 cascade [44, 45]. As an important transcription factor, ZIC1 is essential to the regulation of Hedgehog signaling (Hh), Bone morphogenetic protein (BMP), and Notch signaling pathways in neural development [45, 46]. ZIC1 is also known to be interacting with *GLI* (glioma-associated oncogene homolog) genes,which function as both transcriptional activators and repressors downstream of the Shh signaling pathway and inhibitors of autophagy through the GLI2-PERK-eIF2 axis [47–49]. The hypermethylated signature of *ZIC1* and the fact that ZIC1 is negative regulators of SHH and Notch signaling pathways suggested the potential role of the ZIC1 in the pathogenesis of pediatric CPTs. This hypothesis could be supported by the detection of the notch signaling pathway in the enrichment analysis of DMRs and the hypomethylated signature of all DMPs that annotated to *GLI2* gene in all comparisons. Accordingly, further examining the methylation status of ZICs transcription factors in the CPTs is required to establish their role in the pathogenesis of these tumors.

Proteomic analysis further supported the methylation signature, where most of the DEPs were related to heat shock proteins, ubiquitin, and the proteasome system. Enrichment analysis of both DMPs and DEPs showed similar biological functions, notably unfolded protein response, chaperone-mediated autophagy, late-endosomal microautophagy, protein binding, and cellular response to stress. Aggresomes-positivity expressed the highest number of DEPs and expressed the highest number of enriched pathways. This would suggest that aggresome-based classification reflects a biological fingerprint of CPTs.

The pathogenesis of pediatric CPTs is not well understood and the underlying mechanism of molecular alterations in these tumors remains unknown. In the current study, we examined the proteogenomic signatures of these tumors and identified potential targets that may help to understand these tumors through further analysis. We also defined aggresomes as a mechanism used by CPTs to achieve proteostasis. Aggresomes are thus not only a molecular prognostic marker but also a potential target for treatment of pediatric CPTs.

## Supplementary information

Below is the link to the electronic supplementary material.

Supplementary material 1 (RAR 41089 kb)

Supplementary material 2 (DOCX 2336 kb)

Supplementary material 3 (XLSX 38242 kb)

Supplementary material 4 (XLSX 213 kb)

Supplementary material 5 (XLSX 30 kb)

Supplementary material 6 (XLSX 222 kb)

Supplementary material 7 (XLSX 28 kb)

Supplementary material 8 (XLSX 524 kb)

Supplementary material 9 (XLSX 152 kb)

Supplementary material 10 (XLSX 1941 kb)

## Data Availability

All data generated during this study are included in this article and its supplementary files. Raw methylation data were deposited at the Gene Expression Omnibus (GEO) under accession number GSE156090. The mass spectrometry proteomics data have been deposited to the ProteomeXchange Consortium via the PRIDE [1] partner repository with the dataset identifier PXD021076 and 10.6019/PXD021076.
