# Supplementary material for: Aggresomes predict poor outcomes and implicate proteostasis in the pathogenesis of pediatric choroid plexus tumors
Source: J Neurooncol. 2021 Jan 26;152(1):67–78. doi: 10.1007/s11060-020-03694-3 (PMC7910251; doi:10.1007/s11060-020-03694-3)
Supplement: Supplementary file 2 — Supplementary material 2 (DOCX 2336 kb) [file 11060_2020_3694_MOESM2_ESM.docx]

**Supplementary material**

**Aggresomes predict poor outcomes and implicate proteostasis in the pathogenesis of pediatric choroid plexus tumors**

**Authors**

Nada Amer ^1^, Hala Taha ^2,3^, Dina Hesham ^1^, [Nouran Al-Shehaby](https://www.researchgate.net/profile/Nouran_Al-Shehaby?_sg%5B0%5D=j39xPRFn8Z-JAJ3tmaePtNWY3ttzQkZy2kTRop7Xztv39AKPH5AbwJS7XkdEcBhWkVrFYvg.5TyE9TeSJVnIQqA30X9Lo5YFnZcrs30lgqzl-J3Wyn6DblU8UPk4iY_Zr7MydEDrdlUdzfLigIlxGTG4hq3Tcw&_sg%5B1%5D=ITxspd5vYu0US17diXjutt1-DDNq8nx7YH389d88hci3sFI2XolM-vxVVmlInc1TbLL9Swc.7tLuo8C0QM5C2f2ryvTk-rjpKc_AeFic-29HN1zBY6izPl-L-NaB3DHykFcm3izeYIWpOCTB3XwQL4vpNkEIlQ)^1^, Amal Mosaab ^1,^ Mohamed Soudy^4^, Aya Osama ^4,^ Noura Mahmoud ^5^, Moatasem Elayadi ^5,6^, Ayda Youssef ^7,8^ , Mohamed Elbeltagy ^9,10^ , Mohamed Saad Zaghloul ^11,12^ Sameh Magdeldin^4,13^ , Ahmed A. Sayed ^14,15^  and Shahenda El-Naggar^1*^

1. Tumor Biology Research Program, Basic Research Unit, Research Department, Children’s Cancer Hospital Egypt 57357, Cairo, Egypt.
2. Department of Pathology, Children’s Cancer Hospital Egypt 57357, Cairo, Egypt.
3. Department of Pathology, National Cancer Institute, Cairo University, Cairo, Egypt.
4. Proteomics and Metabolomics Research Program, Basic Research Unit, Research Department, Children’s Cancer Hospital Egypt 57357, Cairo, Egypt.
5. Department of Pediatric Oncology, Children’s Cancer Hospital Egypt 57357, Cairo, Egypt.
6. Department of Pediatric Oncology, National Cancer Institute, Cairo University, Cairo, Egypt.
7. Department of Radiology, Children’s Cancer Hospital Egypt 57357, Cairo, Egypt.
8. Department of Radiology, National Cancer Institute, Cairo University, Cairo, Egypt.
9. Department of Neurosurgery, Children’s Cancer Hospital Egypt 57357, Cairo, Egypt.
10. Department of Neurosurgery, Faculty of Medicine, Cairo University, Cairo, Egypt.
11. Department of Radiotherapy, National Cancer Institute, Cairo University, Cairo, Egypt.
12. Department of Radiotherapy, Children’s Cancer Hospital Egypt 57357, Cairo, Egypt.
13. Department of Physiology, Faculty of Veterinary Medicine, Suez Canal University, Ismailia, Egypt.
14. Genomics Research Program, Basic Research Unit, Research Department, Children’s Cancer Hospital Egypt 57357, Cairo, Egypt.
15. Department of Biochemistry, Faculty of Science, Ain Shams University, Cairo, Egypt.

^*^**Correspondence:** Dr. Shahenda El-Naggar, Tumor Biology Research Program, Basic Research Unit, Research Department, Children’s Cancer Hospital in Egypt 57357, 1 Sekket El Emam, El Madbah El Kadeem Yard, Sayeda Zeinab, Cairo, Egypt

**E-mail:** [shahenda.elnaggar@57357.org](mailto:shahenda.elnaggar@57357.org)

**Phone:** +20 25351500

**ORCID ID:** 0000-0001-6465-9881

**
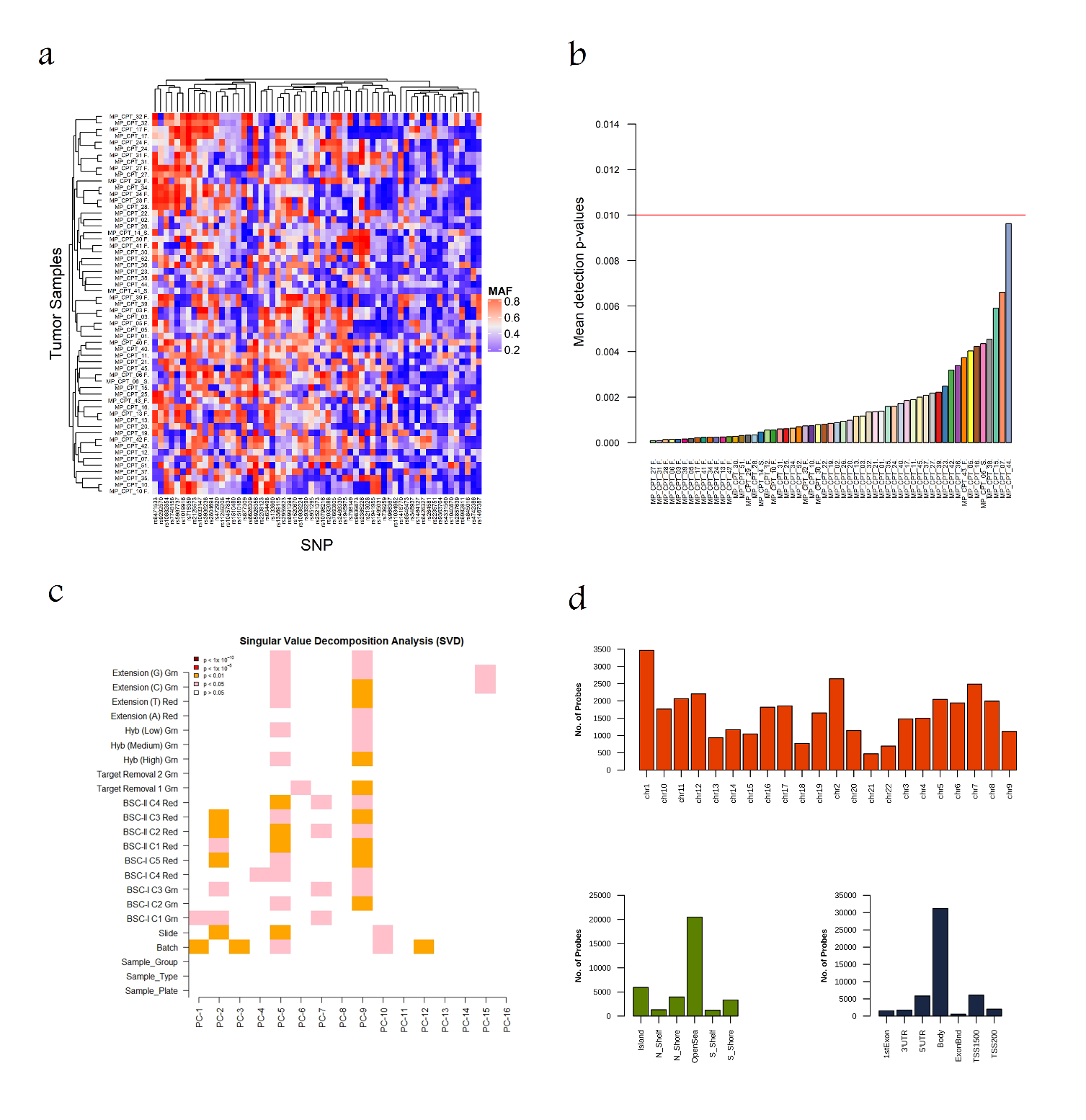
Supplementary Figure 1. Quality control assessment of the Infinium EPIC methylation array**

**Supplementary Figure 1.** Quality control assessment of the Infinium EPIC methylation array. (A) Detection of sample swap of 59 SNPs. (B) Samples with *p-values > 0.01* were excluded from further analysis. (C) Singular value decomposition (SVD) analysis of beta values of CPTs data set. (D) Functional genomic distribution of the most variable methylated CpG sites across all chromosomes and different genomic features.

**Supplementary Figure 2. Methylation-based classification of pediatric CPTs identified two distinct subgroups**


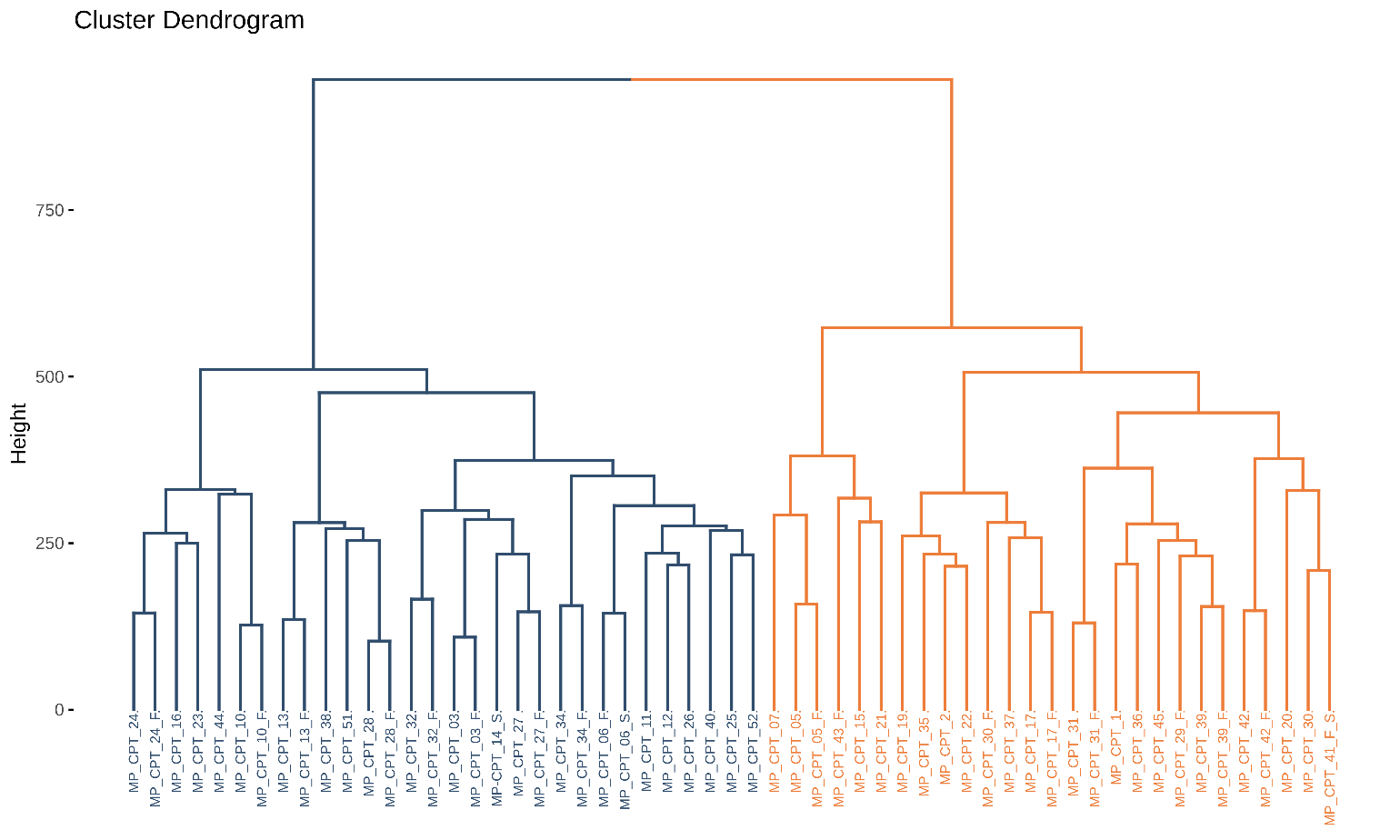


**Supplementary Figure 2.** Methylation-based classification of pediatric CPTs identified two distinct by the hierarchical clustering where cluster "A” represented by blue color and cluster "B" represented by the orange color.

**Supplementary Figure 3. Molecular stratification of pediatric CPTs**

**
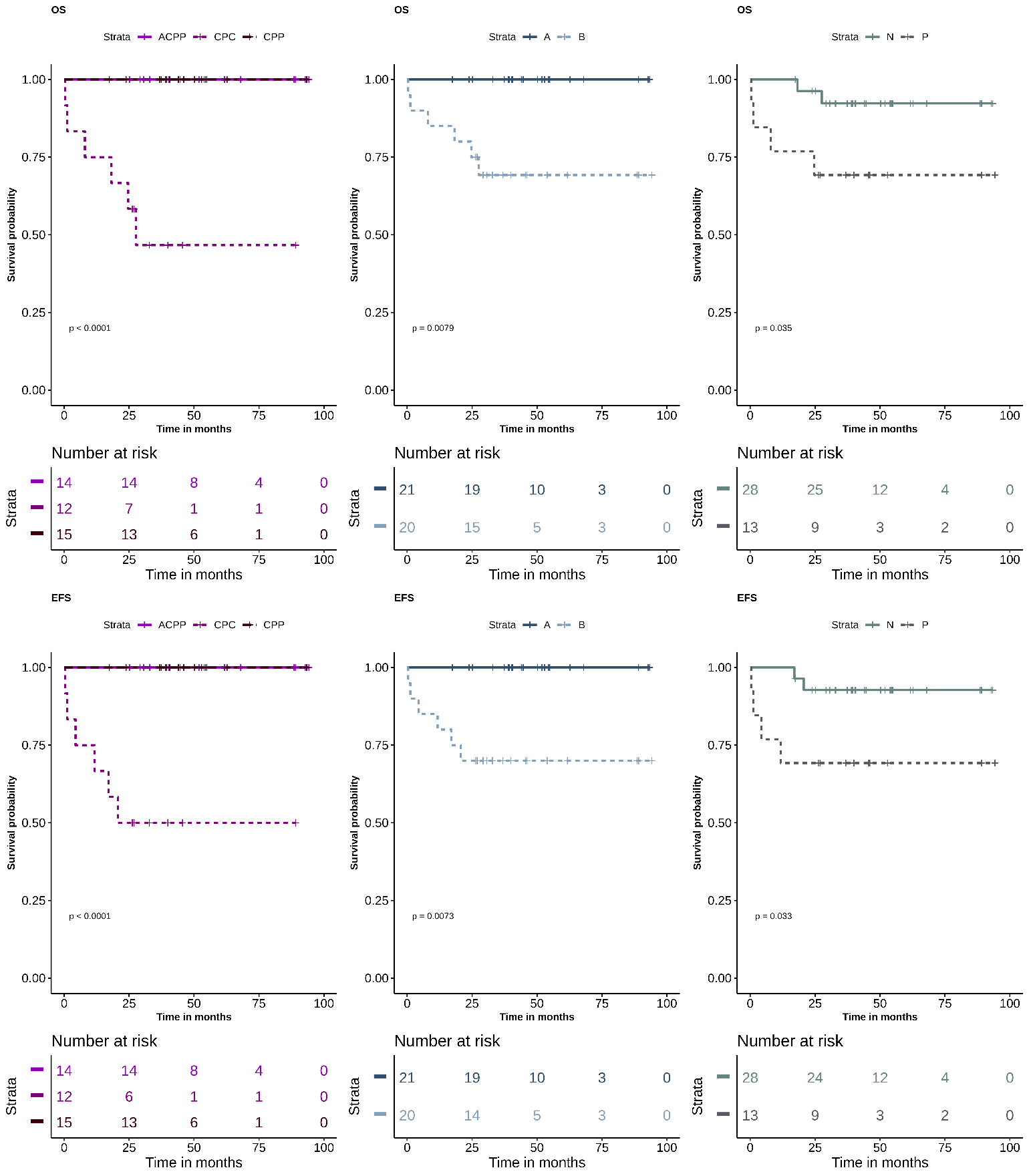
**

**Supplementary Figure 3.** Molecular stratification of pediatric CPTs. Kaplan-Meier plot of overall (OS) and event-free survival (EFS). Survival analysis based on pathological variants (CPC, CPP, and ACPPs), molecular subgroups (B and A clusters), and the status of TP53 (positive and negative TP53 tumors). P-values were calculated using the log-rank test.

**Supplementary Figure 4. Gene ontology enrichment of differentially methylated positions (DMPs)**


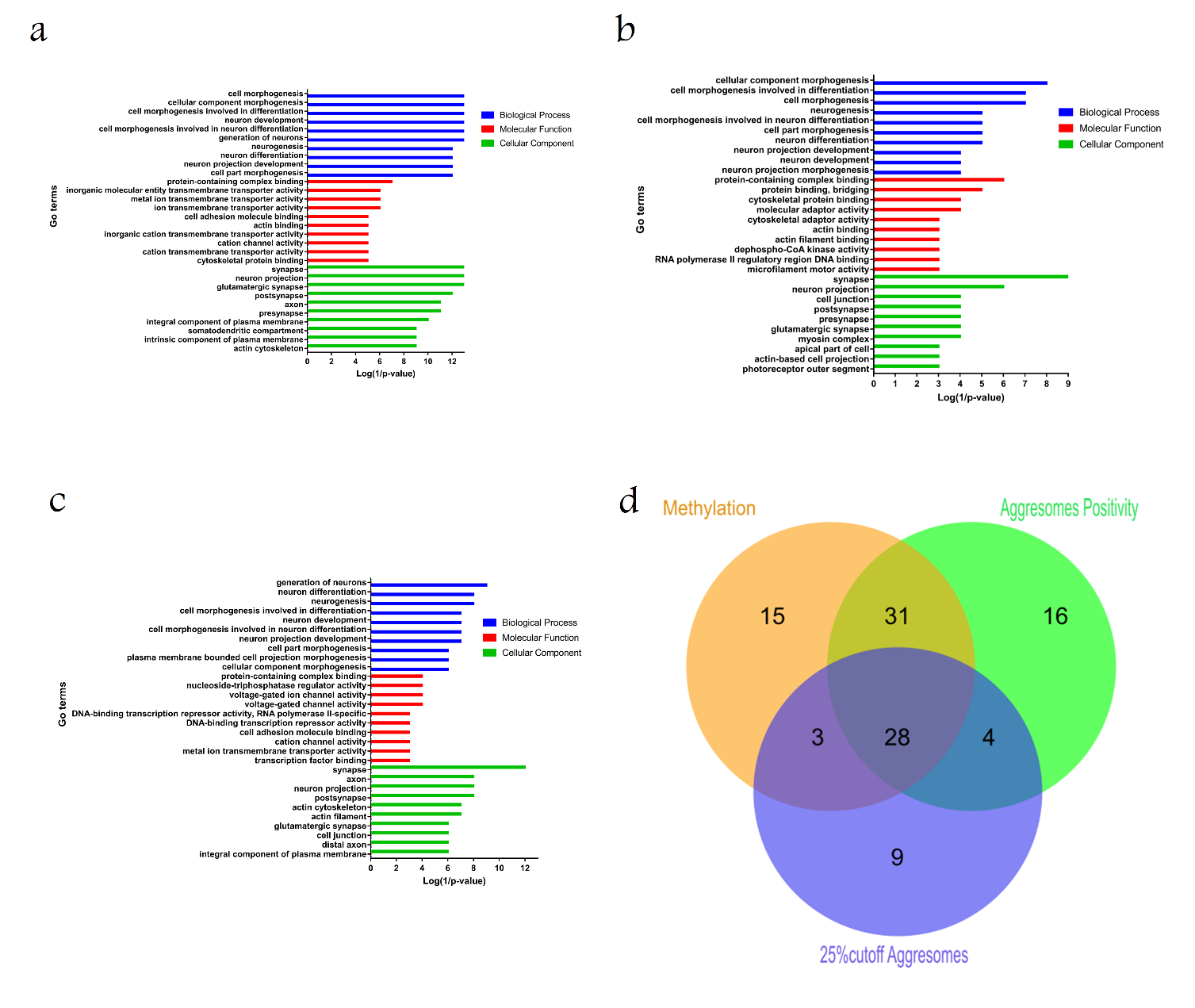


**Supplementary Figure 4.** Gene ontology enrichment of differentially methylated positions (DMPs) showing the Top significant biological processes, molecular functions, and cellular components in (A) methylation-based classification, (B) aggresomes-positivity, and (C) the ≥25% aggresomes cutoff. (D) Venn diagram of common and unique enriched pathways among the three stratification approaches of CPTs

**Supplementary Figure 5. Proteomics analysis of pediatric CPTs**


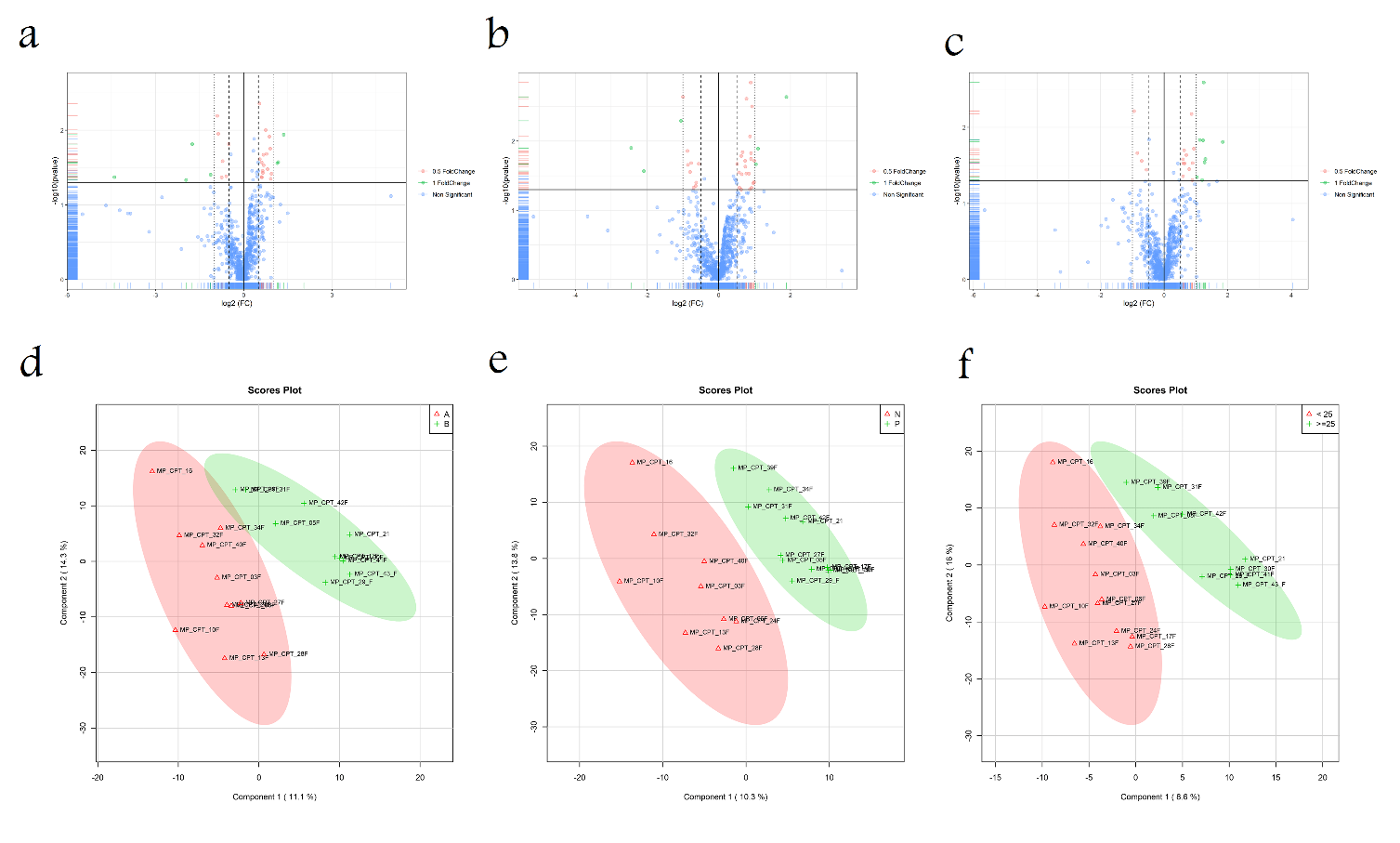


**Supplementary Figure 5.** Proteomics analysis of pediatric CPTs. Volcano plots of differentially expressed proteins (DEPs) in (A) methylation-based classification, (B) aggresomes-positivity, and (C) the ≥25% aggresomes cutoff. The x-axis is the Log2 of fold change and Y-axis is the negative Log10 of the *p-value*. (D) Scores plot for partial least square-discriminant analysis (PLS), where molecular cluster “A” in red and “B” in green. (E) Scores plot for PLS, with aggresomes negative in red and aggresomes positive tumors in green. (F) Scores plot for PLS for tumors with < 25% aggresomes in red and tumors with ≥ 25% aggresomes in the green.

**Supplementary Table 1: Clinicopathological characteristics of CPT patients**

| **Patients Characteristics** | | **Numbers (Percent of total)** | | |
| --- | --- | --- | --- | --- |
| **Gender** | Male | 23 (55%) | | |
|  | Female | 19 (45%) | | |
| **Pathology** | CPC | 12 (28%) | | |
|  | CPP | 15 (36%) | | |
|  | ACPP | 15 (36%) | | |
| **Age Group** | Infant (< 5 years old) | 27 (64%) | | |
|  | From 5 to 14 years old | 14 (33%) | | |
|  | More than 14 years old | 1 (3%) | | |
| **Location of Tumor** | Lateral Ventricle | 31 (74%) | | |
|  | Fourth ventricle | 7 (16%) | | |
|  | Third Ventricle | 2 (5%) | | |
|  | CPA | 2 (5%) | | |
| **Recent Status** | Under Follow Up | 34 (81%) | | |
|  | Dead | 8 (19%) | | |
| **Treatment Protocol** | Follow Up | 26 (62%) | | |
|  | Surgery Only | 3 (7%) | | |
|  | CPT protocol | 13 (31%) | CTH | 6 |
|  |  |  | CTH+RTH | 7 |
| **Status of p53** | Overexpression of p53 | CPC | CPP | ACPP |
|  |  | 7/12 (58.3%) | 1/15 (6.6%) | 2/15 (13.3%) |

Abbreviations: CPC, choroid plexus carcinoma; CPP, choroid plexus papilloma; ACPP, atypical choroid plexus papilloma; CPA, cerebellopontine angle; CTH, chemotherapy; RTH, radiotherapy.

| **ID** | **Gender** | **Pathology** | **TP53** | **Vimentin** | **Cytokeratin** | **Aggresomes** | **Methylation** | **MNP** | **Extent of resection** | **Treatment Protocol** | **CNV_TP53** |
| --- | --- | --- | --- | --- | --- | --- | --- | --- | --- | --- | --- |
| MP_CPT_01 | M | CPC | 60% | 40% | 25% | 40% | B | B (0.94) | GTR | CPT_Protocol | -0.129 |
| MP_CPT_02 | F | ACPP | N | 20% | 3% | 20% | B | B (0.99) | GTR | Follow up | -0.016 |
| MP_CPT_03 | M | ACPP | N | 3% | 1% | N | A | A (0.88) | GTR | Follow up | -0.079 |
| MP_CPT_05 | M | CPC | N | 40% | 40% | 40% | B | B (0.93) | GTR | CPT_Protocol | -0.476 |
| MP_CPT_06 _S | M | CPP | N | 3% | 1% | N | A | A (0.99) | GTR | Follow up | -0.027 |
| MP_CPT_07 | M | CPC | 8% | 20% | 20% | 20% | B | B (0.85) | STR | CPT_Protocol | -0.149 |
| MP_CPT_10 | F | CPP | N | 1% | N | N | A | A (0.88) | GTR | Follow up | 0.041 |
| MP_CPT_11 | M | CPP | N | 3% | N | N | A | A/B (0.49/0.49) | GTR | Follow up | 0.109 |
| MP_CPT_12 | F | CPP | N | 2% | N | N | A | A (0.96) | GTR | Follow up | -0.115 |
| MP_CPT_13 | F | ACPP | N | 1% | N | N | A | A (0.97) | STR | CPT_Protocol | 0.079 |
| MP_CPT_14_S | F | ACPP | N | 20% | 10% | 20% | A | A (0.97) | GTR | Follow up | 0.059 |
| MP_CPT_15 | M | ACPP | N | 25% | 25% | 25% | B | B (0.96) | STR | CPT_Protocol | -0.564 |
| MP_CPT_16 | M | CPP | N | 2% | N | N | A | B (0.86) | GTR | Follow up | -0.216 |
| MP_CPT_17 | M | CPP | 5% | 10% | 7% | 7% | B | B (0.68/0.31) | GTR | Follow up | 0.165 |
| MP_CPT_19 | M | CPC | 80% | 30% | 10% | 30% | B | B (0.98) | GTR | CPT_Protocol | -0.1 |
| MP_CPT_20 | F | CPC | 10% | 50% | 40% | 50% | B | B (0.7) | STR | CPT_Protocol | 0.421 |
| MP_CPT_21 * | F | CPC | N | 60% | N | 60% | B | B (0.99) | GTR | CPT_Protocol | 0.1 |
| MP_CPT_22 | M | CPP | 25% | N | N | 10% | B | B (0.98) | GTR | Follow up | -0.001 |
| MP_CPT_23 | M | CPP | 4% | 2% | N | N | A | B (0.68/0.3) | GTR | Follow up | -0.14 |
| MP_CPT_24 | F | CPP | N | 2% | N | N | A | A/B (0.49/0.49) | GTR | Follow up | -0.477 |
| MP_CPT_25 | F | ACPP | N | 10% | 7% | 10% | A | A (0.97) | GTR | Follow up | 0.165 |
| MP_CPT_26 | F | CPP | N | 1% | N | N | A | A (0.59) | GTR | Follow up | -0.009 |
| MP_CPT_27 | M | CPP | N | 10% | 10% | 10% | A | A (0.7) | GTR | Follow up | -0.102 |
| MP_CPT_28 | M | CPP | N | 5% | 2% | N | A | A (0.98) | GTR | Follow up | 0.127 |
| MP_CPT_29_F | F | CPC | 40% | 90% | 20% | 90% | B | B (0.99) | STR | CPT_Protocol | 0.1 |
| MP_CPT_30 | M | ACPP | N | 30% | 15% | 30% | B | B (0.84) | GTR | Follow up | 0.137 |
| MP_CPT_31 | M | ACPP | N | 30% | 20% | 30% | B | B (0.98) | GTR | Follow up | -0.128 |
| MP_CPT_32 | F | ACPP | N | 5% | N | N | A | A (0.79) | GTR | Follow up | 0.045 |
| MP_CPT_34 | F | CPP | N | 5% | 5% | 15% | A | A (0.94) | GTR | Follow up | 0.171 |
| MP_CPT_35 | M | ACPP | 30% | 15% | 10% | 15% | B | B (0.99) | GTR | Surgery Only | 0.051 |
| MP_CPT_36 | F | CPC | 80% | 30% | 70% | 30% | B | B (0.92) | GTR | CPT_Protocol | 0.0 |
| MP_CPT_37 | M | ACPP | N | 5% | 2% | 5% | B | B (0.63) | GTR | Follow up | 0.243 |
| MP_CPT_38 | M | ACPP | N | 25% | N | 25% | A | B/A (0.31/0.13) | GTR | Follow up | 0.217 |
| MP_CPT_39 | M | CPC | 50% | 40% | 20% | 40% | B | B (0.97) | GTR | CPT_Protocol | -0.252 |
| MP_CPT_40 | M | ACPP | N | 2% | N | N | A | A (0.79) | Biopsy | CPT_Protocol | -0.155 |
| MP_CPT_41 F | M | CPC | N | 60% | 30% | 60% | B | B (0.37) | GTR | CPT_Protocol | -0.163 |
| MP_CPT_42 | F | ACPP | 80% | 25% | 20% | 25% | B | B (0.66/ 0.17) | GTR | Follow up | 0.213 |
| MP_CPT_43_F | F | CPC | 80% | 25% | 30% | 25% | B | B (0.77) | Biopsy | Surgery Only | -0.577 |
| MP_CPT_44 | M | CPP | N | 5% | 6% | 5% | A | B (0.68/0.13) | GTR | Follow up | -0.052 |
| MP_CPT_45 | F | CPC | 70% | 30% | 20% | 35% | B | B (0.98) | GTR | Surgery Only | 0.0 |
| MP_CPT_51 | F | CPP | N | 3% | 10% | N | A | A (0.99) | GTR | Follow up | 0.205 |
| MP_CPT_52 | F | ACPP | N | 2% | N | N | A | A (0.93) | GTR | Follow up | 0.029 |

**Supplementary Table 2: Overview of different classification methods of pediatric CPTs:**

Abbreviations: CPC, choroid plexus carcinoma; CPP, choroid plexus papilloma; ACPP, atypical choroid plexus papilloma, MNP, molecular neuropathology platform; GTR, gross total resection; STR, subtotal resection; CPT_Protocol, choroid plexus tumors protocol (adjuvant chemotherapy and radiotherapy); CNV, copy number variation. * indicates to LFS case, LFS testing is not routinely performed, therefore reporting of one case should not be viewed as a reflection as true percentage in our cohort. Copy number variations (CNV) of TP53 were generated by the MNP platform.

**Supplementary Table 3: Survival analysis for clinical variables:**

| **Patients Characteristics** | | **No. of events** | **OS (2-years)** | **95% CI** | ***p-value*** | **No. of events** | **EFS (2- years)** | **95% CI** | ***p-value*** |
| --- | --- | --- | --- | --- | --- | --- | --- | --- | --- |
| **Pathology** | CPP | 0/15 | 100% | − | < 0.0001 | 0/15 | 100% | − | < 0.0001 |
|  | ACPP | 0/14 | 100% | − |  | 0/14 | 100% | − |  |
|  | CPC | 6/12 | 66.7% | 40 − 93.3% |  | 6/12 | 50% | 21.7 − 78.2% |  |
| **Molecular** | Cluster A | 0/21 | 100% | − | 0.0079 | 0/21 | 100% | − | 0.0073 |
|  | Cluster B | 6/20 | 80% | 62.5 − 97.4% |  | 6/20 | 70% | 50 − 89.9% |  |
| **Aggresomes-positivity** | Negative  Positive | 0/15  6/26 | 100%  84.6% | −  70.6 − 98.5% | 0.057 | 0/15  6/26 | 100%  76.9% | −  60.6 − 93.1% | 0.051 |
| **≥25% aggresomes** | < 25%  ≥ 25% | 0/25  6/16 | 100%  75% | −  53.8 − 96.1% | < 0.001 | 0/25  6/16 | 100%  62.5% | −  38.7 − 86.2% | <0.001 |
| **TP53 status** | < 10 %  ≥ 10% | 2/31  4/10 | 96.7%  70% | 90.2 − 100%  41.5 − 98.4% | 0.035 | 2/31  4/10 | 93.4%  60% | 84.5 − 100%  29.6 − 90.3% | 0.033 |
| **Extent of resection** | GTR  STR/ Biopsy | 5/34  1/7 | 91.1%  85.7% | 81.4 − 100%  59.8 − 100% | 0.97 | 5/34  1/7 | 85.2%  85.7% | 73.2 − 97.1%  59.8 − 100% | 0.97 |
| **Ki-67** | < 30% | 1/32 | 96.7% | 90.2 − 100% | < 0.0001 | 1/32 | 96.9% | 90.8 − 100% | <0.0001 |
|  | ≥ 30% | 5/9 | 55.6% | 23 − 88.1% |  | 5/9 | 44.4% | 11.8 − 76.9% |  |
| **Age** | < 3 years | 4/23 | 86.7% | 72.7 − 100% | 0.53 | 4/23 | 82.6% | 67.1 − 100% | 0.54 |
|  | ≥ 3 years | 2/18 | 94.4% | 83.8− 100% |  | 2/18 | 88.9% | 74.3 − 100% |  |

Abbreviations: CPP, Choroid plexus papilloma; ACPP, Atypical choroid plexus papilloma; CPC, choroid plexus carcinoma; OS, overall survival; CI, confidence interval; EFS, Event free survival; GTR, gross total resection; STR, subtotal resection

**Supplementary Table 4: Univariate analysis of clinical variants**

| **Variant** | **Overall Survival**  **(OS)** | **Event Free Survival**  **(EFS)** |
| --- | --- | --- |
| **Pathology** | p-value < 0.0001 | p-value < 0.0001 |
| **Methylation (B vs. A)** | p-value = 0.0079 | p-value = 0.0073 |
| **Aggresomes (P vs. N)** | p-value = 0.057 | p-value = 0.051 |
| **10% Aggresomes** | p-value = 0.02 | p-value = 0.02 |
| **15% Aggresomes** | p-value = 0.008 | p-value = 0.007 |
| **20% Aggresomes** | p-value = 0.005 | p-value = 0.005 |
| **25% Aggresomes** | p-value < 0.001 | p-value < 0.001 |
| **30% Aggresomes** | p-value = 0.002 | p-value = 0.002 |
| **TP53** | p-value = 0.035 | p-value = 0.033 |

**Supplementary Table 5: Distribution of differentially methylated positions (DMPs):**

| **Stratification approaches** | **Differentially Methylated Positions (DMPs)**  (Adjusted *p-value ≤ 0.05*) | | |
| --- | --- | --- | --- |
|  | **Total number of DMPs** | **Hypomethylated positions** | **Hypermethylated positions** |
| **Methylation-based classification**  Cluster “B” vs. “A” | **65131** | **50447** | **14684** |
| **Aggresomes-positivity**  Aggresomes positive vs. negative | **6859** | **5123** | **1736** |
| **25% cutoff aggresomes**  ≥ 25% aggresomes vs. < 25% | **57819** | **47926** | **9893** |

**Supplementary Table 6:** Differentially methylated positions (DMPs) of the three comparisons.

**Supplementary Table 7:** Enrichment analyses of genes with DMPs of the three comparisons.

**Supplementary Table 8:** Network analysis of genes with DMPs of the three comparisons.

**Supplementary Table 9:** Differentially methylated regions (DMRs) of the three comparisons.

**Supplementary Table 10:** DMR enrichment analysis of the three comparisons.

**Supplementary Table 11:** Filtered and normalized proteomics dataset of the three comparisons.

**Supplementary Table 12:** Differentially expressed proteins (DEPs) of the three comparisons.

**Supplementary Table 13:** Enrichment analyses of DEPs of the three comparisons.
